# Impact of Acetazolamide, a Carbonic Anhydrase Inhibitor, on the Development of Intestinal Polyps in Min Mice

**DOI:** 10.3390/ijms18040851

**Published:** 2017-04-17

**Authors:** Nobuharu Noma, Gen Fujii, Shingo Miyamoto, Masami Komiya, Ruri Nakanishi, Misato Shimura, Sei-ichi Tanuma, Michihiro Mutoh

**Affiliations:** 1 Division of Cancer Prevention Research, National Cancer Center Research Institute, 5-1-1 Tsukiji, Chuo-ku, Tokyo 104-0045, Japan; nnoma1015@gmail.com (N.N.); misato.shimura@gmail.com (M.S.); 2 Division of Biochemistry, Faculty of Pharmaceutical Sciences, Tokyo University of Sciences, 2641 Yamazaki, Noda-shi, Chiba 278-8510, Japan; tanuma@rs.noda.tus.ac.jp; 3 Division of Carcinogenesis and Cancer Prevention, National Cancer Center Research Institute, 5-1-1 Tsukiji, Chuo-ku, Tokyo 104-0045, Japan; gfujii@ncc.go.jp; 4 Epidemiology and Prevention Division, Research Center for Cancer Prevention and Screening, National Cancer Center, 5-1-1 Tsukiji, Chuo-ku, Tokyo 104-0045, Japan; shinmiya@ncc.go.jp (S.M.); mkomiya@ncc.go.jp (M.K.); rnakanis@ncc.go.jp (R.N.)

**Keywords:** carbonic anhydrase, acetazolamide, NRF2, IL-6, colorectal cancer chemoprevention

## Abstract

Colorectal cancer is a common cancer worldwide. Carbonic anhydrase (CA) catalyzes the reversible conversion of carbon dioxide to bicarbonate ion and a proton, and its inhibitor is reported to reduce cancer cell proliferation and induce apoptosis. Therefore, we asked whether acetazolamide, a CA inhibitor, could inhibit intestinal carcinogenesis. Five-week-old male *Apc*-mutant mice, Min mice, were fed a AIN-76A diet containing 200 or 400 ppm acetazolamide. As a result, acetazolamide treatment reduced the total number of intestinal polyps by up to 50% compared to the control group. In addition, the acetazolamide-treated group had low cell proliferation and a high apoptosis ratio in the intestinal polyp epithelial cells. Moreover, the mRNA expression level of proinflammatory cytokines, such as *IL-6*, involved in the cell proliferation was decreased in the polyp part of the acetazolamide-treated group. Next, we examined the effects of acetazolamide on the activation of several transcriptional factors (AP-1, HIF, HSF, NF-κB, NRF2, p53, and STAT3) using a reporter gene assay in human colon cancer cells, Caco-2 cells. Among the examined transcriptional factors, NRF2 transcriptional activation was strongly induced. NRF2-targeting genes, *γGCS*, *GPx1*, *HO-1*, and *NQO-1*, were also elevated in the intestinal polyps of acetazolamide-treated Min mice. Our results suggested that CA is involved in intestinal carcinogenesis. Acetazolamide could inhibit polyp formation through suppressing local/general cytokine levels, i.e., IL-6, via NRF2 activation.

## 1. Introduction

Colorectal cancer (CRC) is a common cancer worldwide. Approximately 1.4 million new CRC cases occurred in 2012, and the incidence is assumed to increase to 2.4 million/year by 2035 [1]. Despite the successive development of anti-cancer agents, the mortality from CRC remains high. Therefore, a new strategy for controlling the development of cancer, such as using chemopreventive agents, is in great demand [2].

Carbonic anhydrase (CA) catalyzes the reversible conversion of carbon dioxide to bicarbonate ion and a proton, and CA is found in many types of organs in humans. This zinc-containing metalloenzyme plays important roles in many physiological processes, including pH and CO_2_ homeostasis and calcification [3]. There are 16 types of CA isozymes, including the cytoplasmic type (CAI, II, III, VII, and XIII); membrane-bound type (CAIV, IX, XII, XIV, and XV); mitochondrial type (CAVa and Vb); and secreted type (CAVI) [3,4]. Some CA isozymes are reported to be associated with carcinogenesis and tumor progression. For instance, two hypoxia-inducible CA isozymes, CAIX and CAXII, promote tumor growth through regulating the pH in the tumor microenvironment [5,6]. CAII is ubiquitously expressed across normal and tumor tissue—only CAIX and CAXII are induced in tumors [7]. It has been reported that CA inhibitors could significantly reduce cell proliferation and induce apoptosis in human cervical cancer HeLa cells and human renal cell adenocarcinoma 786-O cells along with decreasing the intracellular pH [8]. More relevant to the current study, it was recently reported that PEGylated bis-sulfonamides, CA inhibitors, can inhibit the growth of colorectal cancer cells, HT-29 [9].

Acetazolamide is a heterocyclic primary sulfonamide that can inhibit CA. Acetazolamide is an old medication used to treat edema, heart failure, mountain sickness, Meniere’s disease, and more. For cancer cells, it has been reported that acetazolamide could significantly reduce cell proliferation and induce apoptosis in human cancer cells [8]. In an in vivo setting, the administration of acetazolamide (40 mg/kg/d) for 21 days dramatically reduced the number of lung metastases (inhibition rate of lung metastases = 83.9%) in a well-characterized Lewis lung carcinoma model [10]. However, the effects of acetazolamide on intestinal carcinogenesis have not yet been clarified.

Therefore, in the present study, we examined the effects of acetazolamide on intestinal polyp development in *Apc*-mutant Min mice. The Min mouse is an animal model for human familial adenomatous polyposis [11,12]. The mouse has a mutation in codon 850 of the *Apc* gene and resultant activation of β-catenin signaling causes ~100 intestinal polyps (adenoma), mainly in the small intestine. We also examined the effects of acetazolamide on the transcriptional activation of NF-E2-related factor 2 (NRF2) and expression of *B-cell lymphoma 2* (*Bcl-2*). Based on our results, we also discuss the mechanisms involved in the suppressive effects of acetazolamide in Min mice.

## 2. Results

### 2.1. Suppression of Intestinal Polyp Formation in Min Mice by Acetazolamide Treatment

Treatment with 200 (0.6 mg/day/mouse) or 400 ppm (1.2 mg/day/mouse) acetazolamide for 8 weeks to Min mice did not affect the body weight, food intake, or clinical signs compared to untreated mice throughout the experimental period. Table 1 summarizes data for the number and distribution of intestinal polyps in the untreated control (0 ppm) and 200 and 400 ppm acetazolamide-treated groups. Treatment with 200 ppm acetazolamide reduced the total number of polyps to 72.1% of the untreated control value. Of note, the reduction in the middle sections of the intestines was 54.1% (*p* < 0.05) in the acetazolamide group. Treatment with 400 ppm acetazolamide significantly reduced the number of polyps in all parts of the intestine, and the total number of polyps was reduced to 53.7% (*p* < 0.05) of the untreated control value.

**Figure 1:**
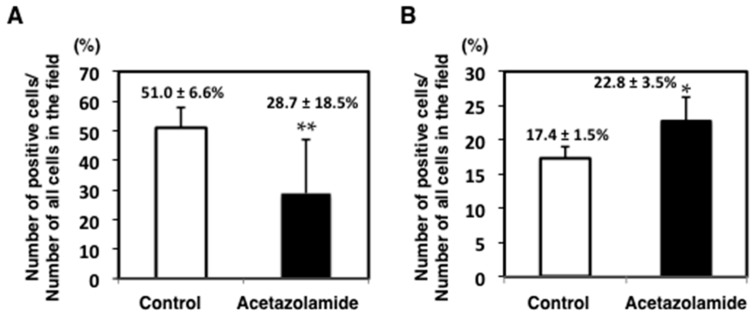
Change in the cell cycle-related and apoptosis-related indexes in intestinal polyps with and without acetazolamide treatment. Immunohistochemistry of proliferating cell nuclear antigen (PCNA) (**A**) and single-stranded DNA (ssDNA) (**B**) was performed in a polyp of the small intestine of a Min mouse treated with 400 ppm of acetazolamide and control treatment. The rates of positive cells were calculated by the number of positive cells over all cells in the field. Data are given as the mean ± SD (No. of polyp = 5). * *p* < 0.05, ** *p* < 0.01 vs. untreated control.

**Figure 2:**
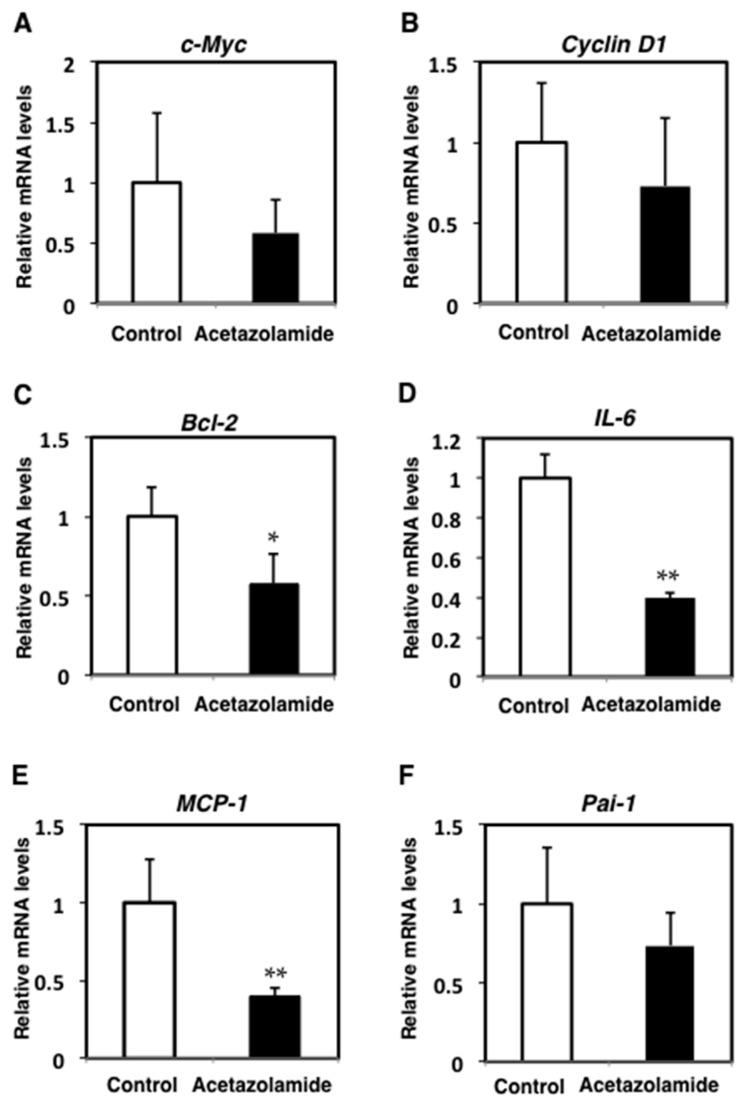
Relative expression levels of cell cycle-related factors, apoptosis-related factors, or proinflammation-related factors in intestinal polyps with and without acetazolamide treatment. Quantitative real-time PCR analyses were performed to determine the *c-Myc* (**A**), *cyclin D1* (**B**), *B-cell lymphoma 2* (*Bcl-2*) (**C**), *interleukine-6* (*IL-6*) (**D**), *monocyte chemoattractant protein-1* (*MCP-1*) (**E**), and *plasminogen activator inhibitor-1* (*Pai-1*) (**F**) mRNA expression levels in the polyps of Min mice with or without 400 ppm of acetazolamide. Values were set at 1.0 in the untreated controls and relative levels are expressed as the mean ± SD (*n* = 5). *Glyceraldehyde-3-phosphate dehydrogenase* (*GAPDH*) mRNA was used to normalize the data. * *p* < 0.05, ** *p* < 0.01 vs. untreated control.

To evaluate the suppressive effects of acetazolamide on intestinal polyp development in Min mice, proliferating cell nuclear antigen (PCNA) was stained in the cell nuclei by immunohistochemistry. The percentage of PCNA-positive cells in each polyp was significantly reduced by acetazolamide treatment from 51.0% (0 ppm) to 28.7% (400 ppm) (Figure 1A). To assess the cell growth inhibition mechanisms in response to acetazolamide, several cell growth-related molecules were analyzed by quantitative RT-PCR. Downregulation of the c-Myc and cyclinD1 expression levels in the small intestinal polyps of Min mice was apparent compared with the untreated group (Figure 2A,B).

To investigate the effect of acetazolamide on apoptosis in intestinal epithelial cells, the intestinal polyps of Min mice were immunohistochemically stained with anti-ssDNA (single-stranded DNA) antibody. The rates for ssDNA positive cells in the intestinal polyps were significantly increased by acetazolamide treatment from 14.7% (0 ppm) to 22.8% (400 ppm) (*p* < 0.05) (Figure 1B). To assess the cell apoptosis induction mechanisms with acetazolamide, antiapoptotic factor *Bcl-2* was analyzed by real-time quantitative reverse transcription polymerase chain reaction (qRT-PCR). The mRNA expression level of *Bcl-2* was decreased to 57.7% (*p* < 0.05) in intestinal polyps compared to the untreated group (Figure 2C).

### 2.2. Suppression of Inflammatory Cytokine mRNA Levels in the Intestinal Polyps and Liver in Min Mice Treated with Acetazolamide

To confirm the expression pattern of acetazolamide targeted molecules in Min mice, the expression levels of the inflammatory cytokines, *interleukine-6* (*IL-6*), *monocyte chemoattractant protein-1* (*MCP-1*), and *plasminogen activator inhibitor-1* (*Pai-1*), were examined by qRT-PCR. The mRNA expression levels of *IL-6* and *MCP-1* were significantly decreased (*p* < 0.01) with the 400 ppm acetazolamide treatment compared to the untreated group (Figure 2D,E). The expression level of *Pai-1* mRNA was decreased to 73.6% with acetazolamide treatment (Figure 2F).

**Figure 3:**
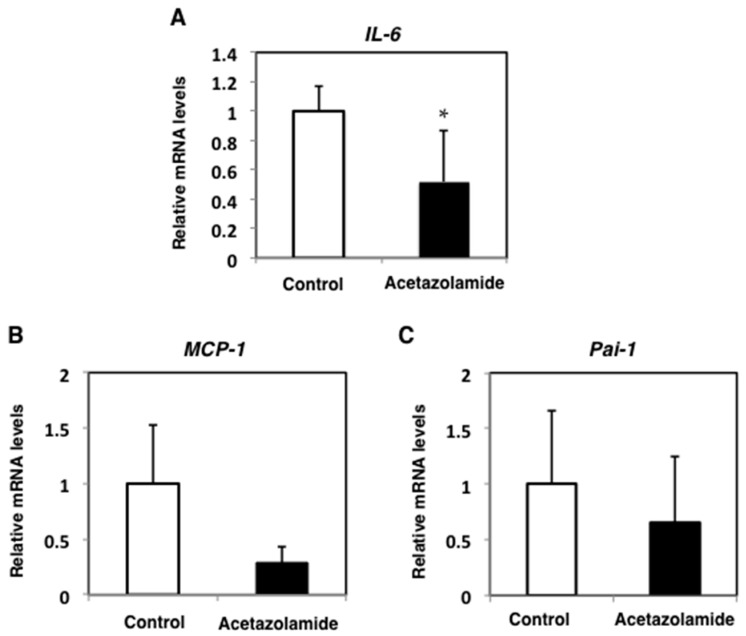
Relative expression levels of inflammatory cytokines *IL-6* (**A**); *MCP-1* (**B**); and *Pai-1* (**C**) in the livers of Min mice with acetazolamide treatment. The data are given as the mean ± SD (*n* = 4). * *p* < 0.05 vs. untreated control.

**Figure 4:**
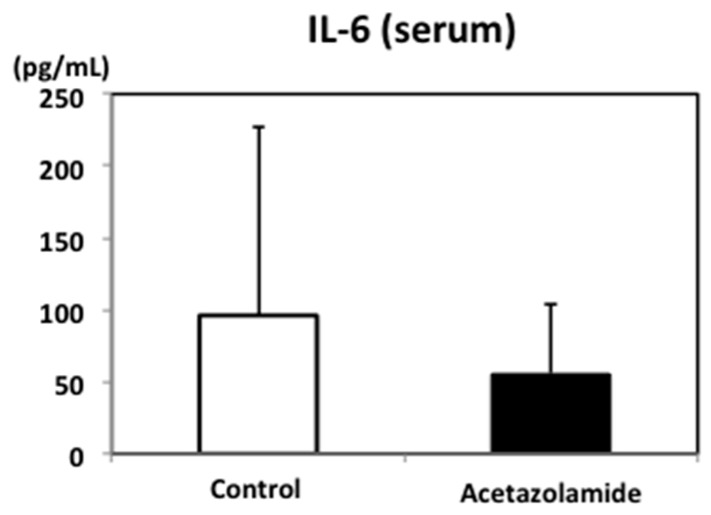
Effects of the serum IL-6 levels in Min mice with acetazolamide treatment. Murine serum IL-6 levels were measured using a Mouse IL-6 Quantikine ELISA Kit (R&D Systems, Minneapolis, MN, USA) according to the manufacturer’s protocol. The data are given as the mean ± SD (*n* = 5).

To investigate the effect of acetazolamide on other organs, the expression levels of hepatic *IL-6*, *MCP-1*, and *Pai-1* in Min mice were examined by qRT-PCR. The mRNA expression levels of *IL-6*, *MCP-1*, and *Pai-1* in the liver were downregulated by 48.9%, 81.4%, and 34.4%, respectively, with the 400 ppm acetazolamide treatment compared to the untreated group (Figure 3). In serum, the IL-6 concentration tended to decrease with acetazolamide treatment (Figure 4).

### 2.3. Effects of Acetazolamide on Seven Oxidative Stress-Related Transcriptional Activities in Human Colorectal Cancer Cells

**Figure 5:**
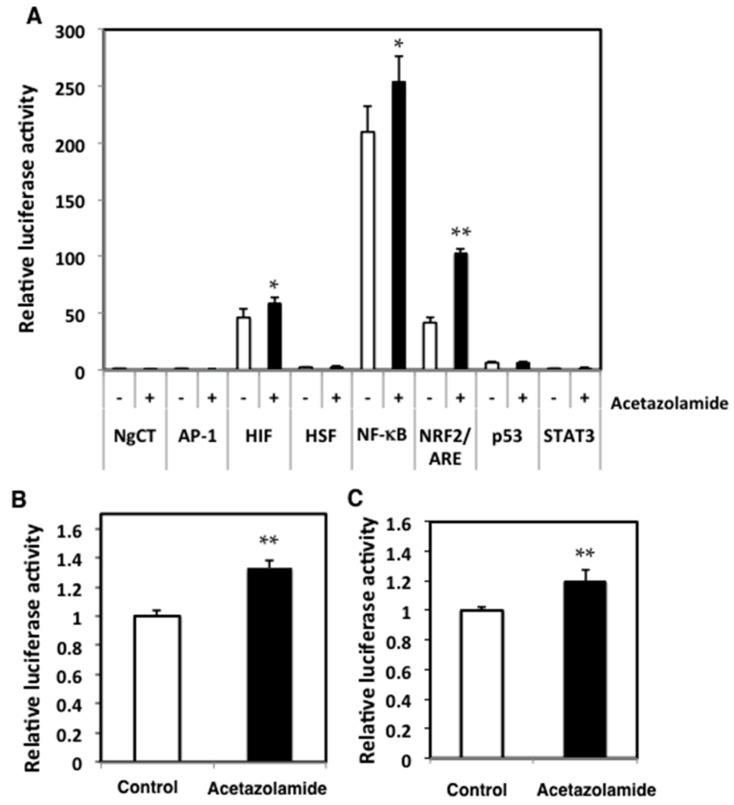
Effects of acetazolamide on the activator protein-1 (AP-1), hypoxia inducible factor (HIF), histamine sensitizing factor (HSF), nuclear factor-κB (NF-κB), NRF2, p53, and signal transducer and activator of transcription 3 (STAT3) transcriptional activity in Caco-2 cells (**A**) with acetazolamide treatment. Values were set at 1.0 in negative control (NgCT). The change in NRF2 transcriptional activity in human colon cancer cell lines, SW48 cells (**B**), and HCT15 cells (**C**), with and without acetazolamide treatment, is shown. The data are given as the mean ± SD (*n* = 3). * *p* < 0.05, ** *p* < 0.01 vs. untreated control.

The effects of acetazolamide on oxidative stress-related transcriptional activity were evaluated in Caco-2 cells. The activator protein-1 (AP-1), hypoxia inducible factor (HIF), histamine sensitizing factor (HSF), nuclear factor-κB (NF-κB), NRF2, p53, and signal transducer and activator of transcription 3 (STAT3) transcriptional activities were tested after 24 h of 500 µM acetazolamide treatment. NRF2 activity was significantly increased by acetazolamide (Figure 5A). These activities were also tested on other human colon cancer cell lines, SW48 and HCT15 cells, and acetazolamide treatment increased the NRF2 activity 1.3- (Figure 5B) and 1.2-fold (Figure 5C), respectively, in these cells.

### 2.4. Effects of the Expression Levels of NRF2-Related Factor mRNA in the Intestinal Polyps

**Figure 6:**
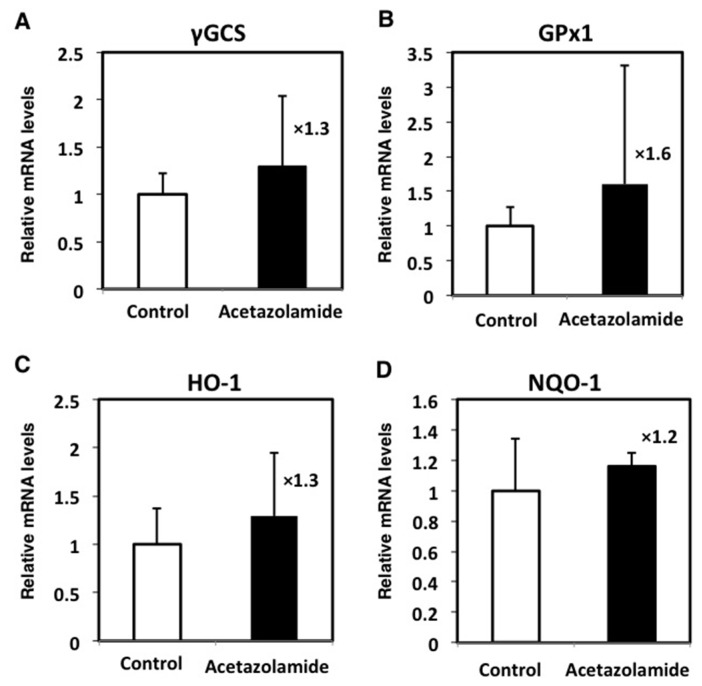
Relative expression levels of NRF2 target genes in intestinal polyps with acetazolamide treatment. The data are given as the mean ± SD (*n* = 5). *γGCS*, *γ-glutamylcysteine synthetase*; *GPx1*, *glutathione peroxidase1*; *HO-1*, *heme oxygenase-1*; *NQO-1*, *NAD(P)H:quinone oxidoreductase-1*. × Means multiplication of relative mRNA levels compare to control.

The expression levels of genes regulated by NRF2 were examined in the intestinal polyps by qRT-PCR. Acetazolamide treatment increased the expression levels of *γ-glutamylcysteine synthetase* (*γGCS*) (1.3-fold), *glutathione peroxidase1* (*GPx1*) (1.6-fold), *heme oxygenase-1* (*HO-1*) (1.3-fold), and *NAD(P)H:quinone oxidoreductase-1* (*NQO-1*) (1.2-fold) (Figure 6).

## 3. Discussion

In the present study, we treated Min mice with acetazolamide and observed a reduction in the number of intestinal polyps compared to the control group. In the intestinal polyps of the acetazolamide-treated group, low cell proliferation and a high apoptosis ratio were observed. The proinflammatory cytokine IL-6 mRNA levels were decreased in the intestinal polyps in acetazolamide-treated Min mice. In addition, a reporter gene assay in human colon cancer cells revealed that NRF2 transcriptional activation was strongly induced by acetazolamide, and other transcriptional factors were similarly affected (AP-1, HIF, HSF, NF-κB, NRF2, p53, and STAT3). Moreover, the expression of NRF2-target genes, *γGCS*, *GPx1*, *HO-1*, and *NQO1*, was elevated in the intestinal polyps of acetazolamide-treated Min mice.

To the best of our knowledge, this is the first report showing that acetazolamide suppressed intestinal polyp formation in mice. As acetazolamide is a pan-CA inhibitor, these data also support the hypothesis that some CA isozymes are involved in intestinal carcinogenesis, and CA could be a good target molecule for cancer chemoprevention.

Our results showed that acetazolamide reduced IL-6 levels in both the local intestinal polyps (i.e., the liver), and the entire mouse (i.e., the serum). IL-6 is a cytokine that is overproduced in chronic inflammation and it could be involved in carcinogenesis. IL-6 activates transcriptional factor STAT3 and upregulates several growth-promoting genes, such as myc and cyclin D1 [13]. In the present study, we observed suppression of *c-Myc* and *cyclin D1* mRNA in the intestinal polyps of Min mice with acetazolamide treatment. Moreover, immunohistochemical staining revealed that acetazolamide treatment reduced the number of PCNA positive cells per field, representing cell growth activity, in the polyps. Therefore, IL-6 reduction is likely involved in the polyp suppressive effects of acetazolamide. In another experiment that we previously performed, IL-6 played an important role in the development of intestinal polyps in Min mice [14,15,16].

NRF2 is a candidate transcriptional factors that may lower the expression levels of IL-6. NRF2 is activated by oxidative stress and binds to the antioxidant response element (ARE) in the promoter region of the genes of cellular antioxidants and phase II enzymes [17,18]. Phase II enzymes include γGCS, GPx1, HO-1, and NQO-1. We demonstrated that acetazolamide activates NRF2 transcriptional activity in human colon cancer cells, Caco-2 cells. Indeed, we confirmed that NRF2-targeting genes, *γGCS*, *GPx1*, *HO-1*, and *NQO-1*, were elevated in the intestinal polyps of Min mice treated with acetazolamide. CA is responsible for regulating the pH level, and acetazolamide decreases the intracellular pH level [8]. Therefore, it is speculated that acetazolamide increased NRF2 activity by inducing oxidative stress through changing the intracellular pH level, but this speculation requires further experimental evidence. Additionally, NRF2 knockout is known to increase the expression of proinflammatory factors, including IL-6 and MCP-1 [19]. Moreover, NRF2 knockout mice are susceptible to hepatic carcinogenesis [20]. These two articles and our results support the evidence that NRF2 is a critical transcriptional factor that regulates inflammation and carcinogenesis.

In polyps from acetazolamide-treated mice, suppression of *Bcl-2* expression and an increased number of ssDNA positive cells per field were observed. The suppressive effect of acetazolamide on *Bcl-2* mRNA expression has not been reported to date. An antiapoptotic protein, Bcl-2, inhibits the release of cytochrome c along with the resultant activation of caspase-9. Caspase-9 cleaves and activates caspase-3, resulting in widespread proteolysis and cell death. Therefore, it is suggested that acetazolamide increases the sensitivity of apoptosis through inhibiting anti-apoptotic Bcl-2.

**Figure 7:**
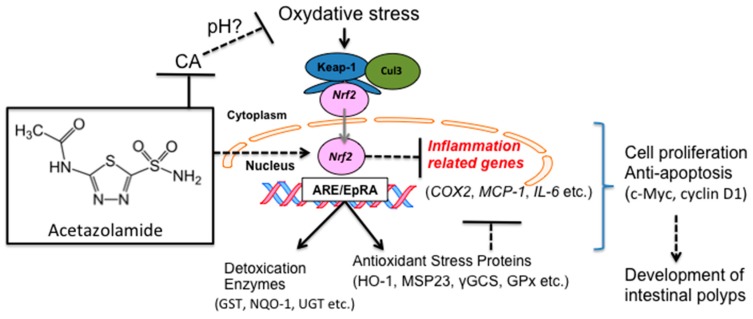
Proposed schema of molecular mechanisms by which acetazolamide suppresses polyp formation in Min mice, partly through the induction of NRF2-transcriptional activity. Solid arrow indicates reliable promoting effect; dotted arrow indicates putative promoting effect; solid T-bar indicates reliable suppressive effect; dotted T-bar indicates putative suppressive effect. Gray arrow indicates nuclear translocation.

In summary, this study suggested that acetazolamide inhibits intestinal polyp formation by inducing apoptosis and inhibiting cell growth in Min mice. Inhibition of cell growth could be partially explained by the activation of NRF2 transcriptional activity, resulting in lower *IL-6* expression (Figure 7). Although there are still unclear mechanisms to explore, acetazolamide could be a candidate chemopreventive agent.

## 4. Materials and Methods

### 4.1. Chemicals

Acetazolamide was purchased from Sigma Chemical Co. (St. Louis, MO, USA).

### 4.2. Cell Culture

SW48 and HCT-15 cells were purchased from the American Type Culture Collection (Manassas, VA, USA). Caco-2 cells were purchased from Sumitomo Dainippon Pharma Co., Ltd. (Osaka, Japan). SW48 and HCT-15 cells were maintained in Dulbecco’s modified eagle medium (DMEM) medium supplemented with 5% heat-inactivated fetal bovine serum (FBS; HyClone Laboratories Inc., Logan, UT, USA) and antibiotics (100 µg/mL streptomycin and 100 U/mL penicillin) at 37 °C, 5% CO_2_. Caco-2 cells were maintained in DMEM medium supplemented with 10% heat-inactivated FBS and antibiotics at 37 °C and 5% CO_2_.

### 4.3. Animals

Male C57BL/6-*Apc*^Min/+^ mice (Min mice) were purchased from The Jackson Laboratory (Bar Harbor, ME, USA). Mice (*n* = 4–5) were housed in a plastic cage with sterilized softwood chips as bedding in a barrier-sustained animal room at 24 ± 2 °C and 55% humidity at a 12-h light/dark cycle. Acetazolamide was mixed at concentrations of 200 and 400 ppm in an AIN-76A powdered basal diet (CLEA Japan, Inc., Tokyo, Japan). We calculated doses of 200 and 400 ppm compared to a human dose. The human dose of acetazolamide is 250–1000 mg/day; therefore, we used acetazolamide at an approximately 1.2–2.4-fold higher dose than the human dose.

### 4.4. Animal Experiment Protocols

**Figure 8:**
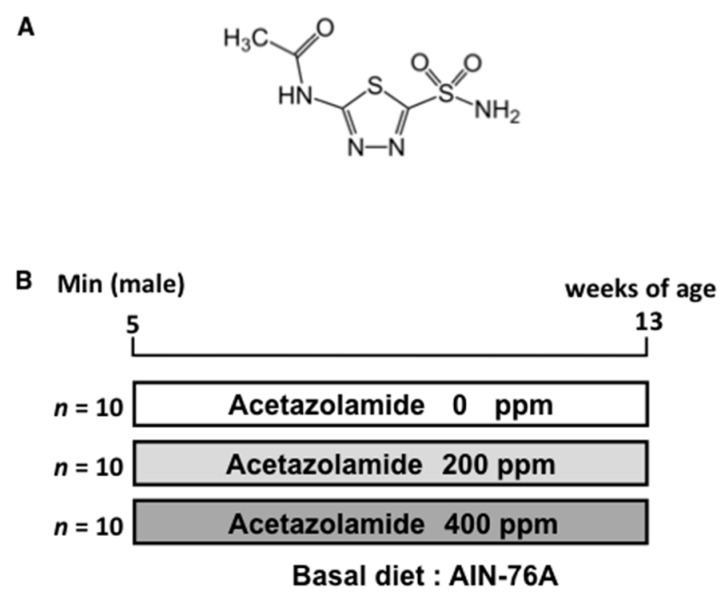
Chemical structure and animal experimental protocol. Structure of acetazolamide (**A**) and experimental protocol (**B**). Ten male Min mice at 5 weeks of age were given 0, 200 (0.6 mg/day/mouse), and 400 (1.2 mg/day/mouse) ppm of acetazolamide for 8 weeks.

Ten male Min mice at 5 weeks of age were given 0, 200, and 400 ppm acetazolamide for 8 weeks (Figure 8). All animals in the same cage were in the same treatment group. Food and water were available ad libitum. The animals were observed daily for clinical signs and mortality. The body weight and food consumption were measured weekly. At the time points for sacrifice, mice were anesthetized, and blood samples were collected from the abdominal vein. The intestinal tract was removed and separated into the small intestine, cecum, and colon. The small intestine was divided into the proximal segment (4 cm in length) and the middle and distal segments, halving the remainder of the proximal segment. Polyps in the proximal segments were counted and evaluated under a stereoscopic microscope; the remaining intestinal mucosa (non-polyp portion) was removed by scraping, and the specimens were stored at −80 °C for quantitative real-time PCR analysis. Other segments were opened longitudinally and fixed flat between sheets of filter paper in 10% buffered formalin. The numbers and sizes of polyps and their distributions in the intestine were assessed with a stereoscopic microscope. The experiments were performed according to the “Guidelines for Animal Experiments in the National Cancer Center” and were approved by the Institutional Ethics Review Committee for Animal Experimentation of the National Cancer Center (permission code: T05-022-C11, approval date: 1 April 2014).

### 4.5. Immunohistochemical Staining of Intestinal Polyps in Min Mice

The small intestines were fixed, embedded, and sectioned as Swiss rolls for further immunohistochemical examination using the avidin-biotin complex immunoperoxidase technique after heating in 10 mM citrate buffer (pH 6.0). The primary antibody was monoclonal mouse anti-PCNA antibody (Calbiochem, La Jolla, CA, USA) or mouse anti-ssDNA antibody (Chemicon, Temecula, CA, USA) at a 100× dilution. The secondary antibody, biotinylated horse anti-mouse IgG (Vector Laboratories, Burlingame, CA, USA), was used at a 200× dilution. Staining was performed using avidin-biotin reagents (Vectastain ABC reagents; Vector Laboratories), 3,3′-diaminobenzidine and hydrogen peroxide, and the sections were counterstained with hematoxylin to facilitate orientation. As a negative control, consecutive sections were immunostained without exposure to the primary antibody. The Swiss roll sections of five mice randomly picked from each group and the total polyps present within one section were evaluated. The ratio of PCNA or ssDNA-positive cells was calculated by the formula % = the number of PCNA positive cells per polyp/the total number of cells in the polyp (100× magnification).

### 4.6. Quantitative Real-Time Polymerase Chain Reaction (PCR) Analyses

Total RNA was isolated from intestinal polyps and non-polyp-containing intestinal mucosa using ISOGEN (Nippon Gene Co., Ltd., Tokyo, Japan); then, it was treated with DNase (Invitrogen, Grand Island, NY, USA) and 1 µg in a final volume, or 20 µL was used for cDNA synthesis using a High Capacity cDNA Reverse Transcription Kit (Applied Biosystems, Foster City, CA, USA). Real-time PCR was conducted using an MJ Research DNA Engine OPTICON 2 System (MJ Research, Inc., Waltham, MA, USA) and SYBR Green Realtime PCR Master Mix (Toyobo Co., Ltd., Osaka, Japan) according to the manufacturer’s instructions. Primer sequences are shown in Table 2. To assess the specificity of each primer set, amplicons generated from the PCR reaction were analyzed for melting curves.

### 4.7. Luciferase Assays for AP-1, HIF, HSF, NF-κB, NRF2, p53, and STAT3 Transcriptional Activity

To measure the AP-1, HIF, HSF, NF-κB, NRF2, p53, and STAT3 transcriptional activity, Caco-2 colon cancer cells were seeded in 96-well plates (1.0 × 10^5^ cells/well). After a 24 h incubation period, the cells were transiently transfected with 100 ng/well of pAP1-Luc, pNF-κB-Luc, pNRF2/ARE-Luc, pp53-Luc, pSTAT3-Luc, or pTA-Luc (Signosis Inc., Santa Clara, CA, USA) reporter plasmid and 10 ng/well pGL4.73 [hRluc/SV40] control plasmid (Promega, Madison, WI, USA) using Lipofectamine 2000 Transfection Reagent (Life Technologies, Inc., Gaithersburg, MD, USA). Transfected cells were cultured for an additional 8 h and treated with 500 µM acetazolamide for 24 h. Then, the firefly and Renilla luciferase activities were determined using the Bright GLO and Renilla GLO Systems (Promega), respectively. The ratio of luciferase activity with each treatment was calculated from the data of triplicate wells with values normalized by the Renilla luciferase activity. In HCT-15 or SW48 cells, the NRF2 activity was measured using the same procedure. The data are expressed as the means ± SD.

### 4.8. Enzyme-Linked Immunosorbent Assay (ELISA) for Measuring Murine Serum IL-6 Levels in Mice

Murine serum IL-6 levels were measured using a Mouse IL-6 Quantikine ELISA Kit (R&D Systems, Minneapolis, MN, USA) according to the manufacturer’s protocol.

### 4.9. Statistical Analyses

All results are expressed as the means ± SD values, and statistical analyses were performed using Student’s *t*-tests. The exceptions are the examination of the body weight, diet intake, organ weight, and intestinal polyp formation/size distribution, which were analyzed by Bonferroni’s test. Differences were considered statistically significant at * *p* < 0.05 and ** *p* < 0.01.

## Figures and Tables

**Table 1:** The number of intestinal polyps/mouse in Min mice treated with or without acetazolamide.

Dose (ppm)	Number of Mice	Number of Polyps/Mouse				
		Small Intestine			Colon	Total
		Proximal	Middle	Distal		
0	10	4.5 ± 1.5	17.0 ± 9.7	26.8 ± 9.8	0.5 ± 1.0	48.8 ± 18.8
200	10	3.9 ± 1.8	9.2 ± 3.2 *	21.8 ± 6.3	0.3 ± 0.7	35.2 ± 9.2
400	10	2.7 ± 1.2 *	8.0 ± 2.3 *	15.4 ± 6.2 **	0.1 ± 0.3	26.2 ± 7.8 **

Data are presented as the means ± SD. Significantly different from the untreated control group at * *p* < 0.05 and ** *p* < 0.01.

**Table 2:** Primers for mice.

Gene		Sequence
*c-Myc*	Forward	GCT CGC CCA AAT CCT GTA CCC T
	Reverse	TCT CCA CAG ACA CCA CAT CAA TTT C
*Cyclin D1*	Forward	TGA CTG CCG AGA AGT TGT GC
	Reverse	CTC ATC CGC CTC TGG CAT T
*Bcl-2*	Forward	ACT TCG CAG AGA TGT CCA GTC A
	Reverse	TGG CAA AGC GTC CCC TC
*IL-6*	Forward	TGT TCT CTG GGA AAT TCG TGG A
	Reverse	AAG TGC ATC ATC GTT GTT CAT ACA
*MCP-1*	Forward	CAG CCA GAT GCA GTT AAC GC
	Reverse	GCC TAC TCA TTG GGA TCA TCT TG
*Pai-1*	Forward	ACG TTG TGG AAC TGC CCT AC
	Reverse	GCC AGG GTT GCA CTA AAC AT
*γGCS*	Forward	CTA CCA CGC AGT CAA GGA CC
	Reverse	CCT CCA TTC AGT AAC AAC TGG
*GPx1*	Forward	AAT GTC GCG TCT CTC TGA GG
	Reverse	TCC GAA CTG ATT GCA CGG G
*HO-1*	Forward	GAT AGA GCG CAA CAA GCA GAA
	Reverse	CAG TGA GGC CCA TAC CAG AAG
*NQO-1*	Forward	AGG ATG GGA GGT ACT CGA ATC
	Reverse	TGC TAG AGA TGA CTC GGA AGG

## Abbreviations

AP-1	Activator protein-1
ARE	Antioxidant response element
Bcl-2	B-cell lymphoma 2
CA	Carbonic anhydrase
CRC	Colorectal cancer
FBS	Fetal bovine serum
γGCS	γ-Glutamylcysteine synthetase
GAPDH	Glyceraldehyde-3-phosphate dehydrogenase
GPx1	Glutathione peroxidase 1
HIF	Hypoxia inducible factor
HNPCC	Hereditary nonpolyposis colorectal cancer
HO-1	Heme oxygenase-1
HSF	Histamine sensitizing factor
IL-6	Interleukine-6
MCP-1	Monocyte chemoattractant protein-1
NF-κB	Nuclear factor-κB
NQO-1	NAD(P)H:quinone oxidoreductase-1
NRF2	NF-E2-related factor 2
Pai-1	Plasminogen activator inhibitor-1
PCNA	Proliferating cell nuclear antigen
PCR	Polymerase chain reaction
ssDNA	Single-stranded DNA
STAT3	Signal transducer and activator of transcription 3
